# Quantitative CT screening improved lumbar BMD evaluation in older patients compared to dual-energy X-ray absorptiometry

**DOI:** 10.1186/s12877-023-03963-6

**Published:** 2023-04-17

**Authors:** Wentao Lin, Chaoqin He, Faqin Xie, Tao Chen, Guanghao Zheng, Houjie Yin, Haixiong Chen, Zhiyun Wang

**Affiliations:** 1 Department of Spine Surgery, Shunde Hospital, Southern Medical University, The First People’s Hospital of Shunde Foshan), Foshan, Guangdong China; 2 The Second Clinical Medical College of Southern Medical University, Guangzhou, Guangdong China; 3 Department of Radiology and Image, Shunde Hospital, Southern Medical University, The First People’s Hospital of Shunde Foshan), Foshan, Guangdong China

**Keywords:** Bone mineral density, Vertebral fracture, Lumbar osteoporosis, Quantitative computed tomography, Dual X-ray absorptiometry

## Abstract

**Background:**

Robust evidence on whether diagnostic discordance exists between lumbar osteoporosis detected by quantitative computed tomography (QCT) vs. dual-energy X-ray absorptiometry (DXA) is still lacking. In this study involving a relatively large prospective cohort of older men (aged > 60 years) and postmenopausal women, we assessed lumbar QCT-derived volumetric bone mineral density (vBMD) and DXA-derived area BMD and evaluated their predictive performance for prevalent vertebral fracture (VF).

**Methods:**

A total of 501 patients who underwent spinal surgery from September 2020 to September 2022 were enrolled. The criteria recommended by the American College of Radiology and the World Health Organization were used for lumbar osteoporosis diagnosis. The osteoporosis detection rates between QCT and DXA were compared. QCT-vBMD was plotted against the DXA T score, and the line of best fit was calculated based on linear regression. Multivariate logistic regression was used to analyze the associations between risk factors and VF. Receiver operating characteristic curve analysis was performed, and the corresponding area under the curve (AUC) was calculated.

**Results:**

QCT screening showed that 60.7% of patients had osteoporosis, whereas DXA screening showed that 50.7% of patients had osteoporosis. Diagnoses were concordant for 325 (64.9%) patients. In all, 205 patients suffered a VF of at least one anatomic level. Of these, 84.4% (173/205) were diagnosed with osteoporosis by QCT, while only 73.2% (150/205) were diagnosed by DXA. Multivariate logistic regression showed that osteoporosis detected by QCT exhibited a stronger relationship with VF than that detected by DXA (unadjusted OR, 6.81 vs. 5.04; adjusted OR, 3.44 vs. 2.66). For discrimination between patients with and without VF, QCT-vBMD (AUC = 0.802) showed better performance than DXA T score (AUC = 0.76).

**Conclusion:**

In older patients undergoing spinal surgery, QCT-vBMD is more helpful than DXA in terms of osteoporosis detection rate and prediction of patients with prevalent VFs.

**Supplementary Information:**

The online version contains supplementary material available at 10.1186/s12877-023-03963-6.

## Background

Osteoporosis is a prevalent skeletal disorder characterized by bone mass loss and microarchitectural deterioration, leading to fragility and fracture. Although bone strength is multifactorial, the measurement of bone mineral density (BMD) plays a pivotal role and has been widely used in clinical practice due to its availability and affordability [1]. Currently, the most commonly used BMD measurement methods include dual X-ray absorptiometry (DXA) and quantitative computed tomography (QCT). The former provides a measurement of the area BMD (aBMD) in two dimensions, while the latter allows for quantification of the volumetric BMD (vBMD) of trabecular bone and avoids interference from aortic calcifications, bone spur formation, and abdominal fat [2]. As spinal BMD can provide useful information for surgical planning and decision-making, it is of greater clinical value to focus on BMD evaluation for patients about to undergo spinal surgery [3–6].

The diagnostic performance of spinal osteoporosis by QCT and DXA have been compared in several studies, including cross-sectional studies and case‒control studies, but their results have been discordant [7–11]. There are several factors that contribute to these discrepancies in results. Studies performed in small samples may lack statistical power [7]. A high prevalence of osteoporosis was identified in elderly patients undergoing spine surgery. Thus, the results obtained from populations with a relatively low prevalence of osteoporosis or young women with type I diabetes mellitus may not be generalizable to the specific population undergoing spine surgery [8–10]. Using the same diagnostic category may contribute to the comparability of data but cause serious deterioration in the accuracy of the procedures [11, 12]. It is inappropriate to use a threshold of T score = -2.5 for both QCT and DXA [13].

Various established risk factors are associated with bone mass loss, including smoking, older age, female sex, body mass index, and diabetes mellitus [14–16]. Meanwhile, osteoporosis is still the most sensitive predictor for fragility fractures [17]. To date, robust evidence on whether a diagnostic discordance exists between spinal osteoporosis detected by QCT and that detected by DXA remains lacking. Additional studies designed to assess spinal BMD by QCT and DXA are needed to examine these potential confounders and mediators of the association as well as the clinical outcomes.

Therefore, we assessed spinal QCT-vBMD and DXA-aBMD in a large prospective cohort of older men (aged > 60 years) and postmenopausal women and evaluated the predictive performance regarding vertebral fracture (VF).

## Methods

### Study design and population cohort

This study was approved by the local institutional review board (IRB) and conducted in accordance with the tenets of the Declaration of Helsinki. Older patients who visited the Spine Surgery Department of a single medical institution and were about to undergo spine surgery were enrolled in this study. Included patients’ demographic information was recorded at admission, including sex, age, BMI, smoking status, medication history, primary diagnosis, and comorbidities. All participants were scheduled for QCT and DXA examinations as well as blood testing for serological indicators within a week of their admission; they were not permitted to receive any antiosteoporotic treatment during this time except for calcium and vitamin D. Those with a history of spinal instrumentation surgery, a history of severe trauma, spinal tumors, spinal infection, and severe spinal deformity were excluded.

### BMD evaluation and diagnostic category

An Aquilion 64-slice CT scanner (Toshiba Medical System Inc., Tokyo, Japan) with the Mindways QCT pro system (Mindways Software Inc., Austin, TX) was used for acquiring QCT image sequences and was asynchronously calibrated by a Model 4 calibration phantom that allowed for individuals’ BMD evaluations at a different time. The method of region of interest (ROI) selection has previously been described in detail [2]. Satisfying the point of caution involves maximizing the ROI while excluding the basal vertebral vein, cortical bone, and sclerotic regions. According to the manufacturer’s protocols, standard QCT measurements were utilized to evaluate BMD at the L1-L2 vertebrae. For the individuals in whom the ROI could not be measured at the L1-L2 levels, the adjacent vertebral body was used as a surrogate for measurement of vBMD. The diagnostic thresholds at the L1-L2 levels recommended by the American College of Radiology were used for lumbar osteoporotic diagnosis (normal, vBMD > 120 mg/cm^3^; osteopenia, 80 mg/cm^3^ ≤ vBMD ≤ 120 mg/cm^3^; osteoporosis, vBMD < 80 mg/cm^3^) [18]. The presence of vertebral fractures was assessed on sagittal CT images by applying the Genant semiquantitative visual approach.

GE Lunar scanners (GE Lunar Prodigy) and DAX Brovo DXA scanners (GE Healthcare, WI, USA) were used to obtain DXA-aBMD at the L1–L4 levels by a well-trained radiologist blinded to the study. The aBMD was then presented as the T score calculated using the following formula:

$$T\;score=\hspace{0.17em}(\mathrm{mea}s\mathrm{ure}\;\mathrm{value}-\mathrm{peak}\;\mathrm{aBMD})/\mathrm{standard}\;\mathrm{deviation}\;\mathrm{of}\;\mathrm{aBMD}\;\mathrm{in}\;\mathrm{normal}\;\mathrm{adults}$$

Quality assurance and quality control were previously described [2]. The diagnostic thresholds at the L1-L4 levels recommended by the World Health Organization (WHO) were used for diagnostic category (normal, -1.0 or above; osteopenia, between -1.0 and -2.5; osteoporosis, -2.5 or below). The possible difference in osteoporotic category between DXA and QCT was classified as a major or minor discordance [19]. The former indicates that the patient was diagnosed with osteopenia by one technique but osteoporosis or normal by the other, while the latter means the patient was diagnosed with osteoporosis by one technique but normal BMD by the other. In the study, all DXA and QCT scans were evaluated by the same experienced radiologist who was blinded to the study.

### Statistical analysis

The normality of the data distribution was tested with the Kolmogorov‒Smirnov normality test. Continuous and categorical variables are expressed as the mean ± standard deviation and frequencies and percentages, respectively. The baseline characteristics between two groups were evaluated using one-way ANOVA if the homogeneity of variance requirement was met; otherwise, the rank sum test was used. Chi-square tests were used for comparison of proportions, which were adjusted using the Bonferroni correction for multiple pairwise comparisons. QCT-vBMD was plotted against the DXA T score, followed by calculation of the line of best fit based on linear regression. Contextually, a residual analysis, a “residuals versus fits plot” was conducted. It is a scatter plot of residuals on the y-axis and fitted values (estimated responses) on the x-axis. The plot was used to detect nonlinearity, unequal error variances, and outliers. Subgroup analysis of patient sex, age stratification in postmenopausal women, and presence of VF was performed similarly. Multivariate logistic regression was used to analyze the associations between risk factors and VF, and the odds ratio (OR) and 95% confidence interval (CI) of osteoporosis detected by QCT and DXA were calculated. Variables were transformed and standardized using z scores. Collinearity was assessed with variance inflation factors (VIF). Receiver operating characteristic (ROC) curve analysis was performed to estimate the VF diagnostic performance of the QCT-vBMD and DXA T scores, from which the corresponding area under the curve (AUC) was calculated. SPSS 25.0 (IBM Inc., Armonk, NY, USA), Origin 2021 (Origin Lab Corporation, Northampton, MA, USA) and GraphPad Prism 8 (GraphPad Software Inc., San Diego, CA, USA) were used for statistical analysis and production of all graphs and dot plots. Statistical significance was set at p < 0.05.

## Results

**Fig. 1:**
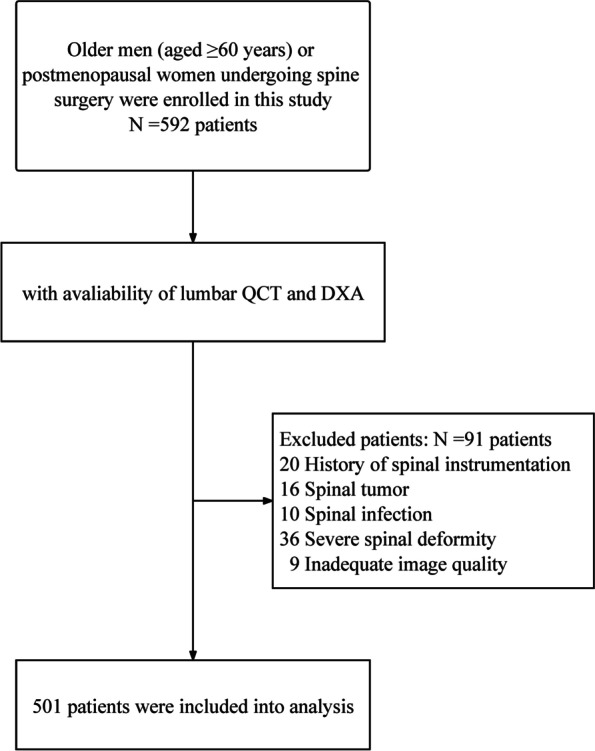
Flow chart of patient enrollment

The final sample comprised 501 participants (395 women and 106 men; mean age: 71.3 ± 7.2 and 67.6 ± 10.4 years, respectively), and the flowchart of participants is shown in Fig. 1. The mean BMI of women and men was 23.8 ± 3.6 and 23.7 ± 3.6 kg/m^2^, respectively. A comparison of baseline characteristics between male and female patients is shown in Table 1. In this cohort, the most frequent primary diagnosis was VF (38.3%), followed by lumbar disc herniation (36.9%). Cardiovascular disease (46.3%), such as hypertension and coronary heart disease, was the most widely reported comorbidity, followed by diabetes mellitus (15.6%). The proportion of elderly men with spinal osteoporosis was significantly lower than that of postmenopausal women (25.5% vs. 57.5% according to DXA category and 44.3% vs. 65.1% according to QCT category, *P* < 0.01).

**Table 1:** Baseline characteristics of study participants

**Variables**^a^	**Total (*N* = 501)**	**Male (*N* = 106)**	**Female (*N* = 395)**	***P* value**
Age, year	68.3 ± 9.9	71.3 ± 7.2	67.6 ± 10.4	**0.001**
Weight, kg	57.3 ± 10.2	63.8 ± 10.5	55.6 ± 9.4	**0.001**
Height, cm	155.4 ± 7.4	163.8 ± 6.4	153.1 ± 5.9	**0.001**
Body mass index, kg/m^2^	23.7 ± 3.6	23.8 ± 3.6	23.7 ± 3.6	0.852
Serum ionized calcium, mmol/L	2.29 ± 0.14	2.27 ± 0.14	2.30 ± 0.14	0.075
Serum phosphorus, mmol/L	1.12 ± 0.17	1.05 ± 0.19	1.13 ± 0.17	**0.001**
Serum uric acid, umol/L	308.9 ± 100.4	336.6 ± 110.3	301.2 ± 96.3	**0.003**
Creatinine clearance, ml/min	66.3 ± 23.1	62.6 ± 22.6	67.3 ± 23.1	0.063
25‐hydroxyvitamin D, ng/mL	28.9 ± 11.2	35.4 ± 14.6	27.2 ± 9.3	**0.001**
Parathyroid hormone, pg/mL	45.0 ± 20.7	41.6 ± 22.2	46.0 ± 20.1	0.074
Calcitonin, pg/mL	0.96 ± 1.45	2.04 ± 2.67	0.67 ± 0.59	**0.001**
Alkaline phosphatase, U/L	78.6 ± 27.9	81.3 ± 36.2	77.9 ± 25.2	0.371
P1NP, ug/L	71.3 ± 35.7	62.4 ± 33.2	73.6 ± 36.1	0.061
CTX-1, ug/L	0.29 ± 0.27	0.27 ± 0.25	0.29 ± 0.27	0.615
Hemoglobin A1c, %	6.2 ± 1.1	6.1 ± 0.9	6.2 ± 1.1	0.274
Smoking status, n (%)	17 (3.4)	16 (15.1)	1 (0.3)	**0.001**
Steroid use, n (%)	21 (4.2)	7 (6.6)	14 (3.5)	0.163
DXA category				
Normal, n (%)	109 (21.8)	45 (42.5)	64 (16.2)	**0.001**^#^
Osteopenia, n (%)	138 (27.5)	34 (32.1)	104 (26.3)	0.905
Osteoporosis, n (%)	254 (50.7)	27 (25.5)	227 (57.5)	**0.001**^#^
QCT category				
Normal, n (%)	45 (9)	11 (10.4)	34 (8.6)	0.578
Osteopenia, n (%)	152 (30.3)	48 (45.3)	104 (26.3)	**0.001**^#^
Osteoporosis, n (%)	60.7 (60.7)	47 (44.3)	257 (65.1)	**0.001**^#^
Comorbidities, n (%)				
Cardiovascular disease	229 (46.3)	59 (55.7)	170 (43.0)	
Diabetes mellitus	78 (15.6)	22 (20.8)	56 (14.2)	
Respiratory disease	38 (7.6)	18 (17)	20 (5.1)	
Malignancy	30 (6.0)	10 (9.4)	20 (5.1)	
Cerebrovascular disease	21 (4.2)	9 (8.5)	12 (3.0)	
Chronic kidney disease	18 (3.6)	6 (5.7)	12 (3.0)	
Primary diagnosis, n (%)				
Vertebral compression fracture	192 (38.3)	30 (28.3)	162 (41)	
Lumbar disc herniation	185 (36.9)	45 (42.5)	140 (35.4)	
Lumbar spinal stenosis	49 (9.8)	15 (14.2)	34 (8.6)	
Spondylolysis/Spondylolisthesis	37 (7.4)	8 (7.5)	29 (7.3)	
Lumbar degenerative scoliosis	21 (4.2)	3 (2.8)	18 (3.6)	
Other	17 (3.4)	5 (5.7)	12 (3.0)	

*DXA* dual x-ray absorptiometry, *QCT* quantitative computed tomography, *P1NP* procollagen-1 N-terminal peptide, *CTX-1* C-terminal telopeptide of type-1 collagen

^a^Continuous variables were expressed as means and standard deviation and tested for statistical significance with Students t-test. Categorical variables were expressed as counts and frequencies and were tested with chi-squared test, and ^#^Bonferroni correction was used for multiple pairwise comparisons. Bold values indicate a statistically significant difference between male and female patients (*P* < 0.05)

### Discordance in osteoporosis diagnoses between QCT and DXA

In this cohort, QCT screening showed that 60.7% had osteoporosis, 30.3% had osteopenia, and 9% had normal BMD, whereas DXA screening showed that 50.7% of patients had osteoporosis, 27.5% had osteopenia, and 21.8% had normal BMD. Diagnoses were concordant for 325 (64.9%) patients. Of the other 176 patients with diagnostic discordance, 14 (2.8%) were major and 162 (32.3%) were minor. A total of 83 (16.5%) patients met the criteria for osteoporosis via QCT but were diagnosed with osteopenia or normal BMD according to the DXA criteria, while 33 (6.6%) were diagnosed with osteoporosis by DXA but not by QCT (Table 2). Similar results of the distribution of diagnostic category for QCT-vBMD and DXA T score were also obtained in the subgroup analysis of male and female patients (Supplementary Tables 1 and 2).

**Table 2:** Distribution of diagnostic category for lumbar BMD

		QCT			
		Normal	Osteopenia	Osteoporosis	Total
DXA	Normal	**40 (8.0%)**	55 (11%) ^a^	14 (2.8%) ^b^	109 (21.8%)
	Osteopenia	5 (1%) ^a^	**64 (12.8%)**	69 (13.7%) ^a^	138 (27.5%)
	Osteoporosis	0^b^	33 (6.6%) ^a^	**221 (44.1%)**	254 (50.7%)
	Total	45 (9.0%)	152 (30.3%)	304 (60.7%)	501 (100%)

*BMD* bone mineral density, *DXA* dual x-ray absorptiometry, *QCT* quantitative computed tomography

^a^minor discordance; ^b^major discordance; boldface indicates diagnostic concordance

### BMD distribution and subgroup analysis

**Fig. 2:**
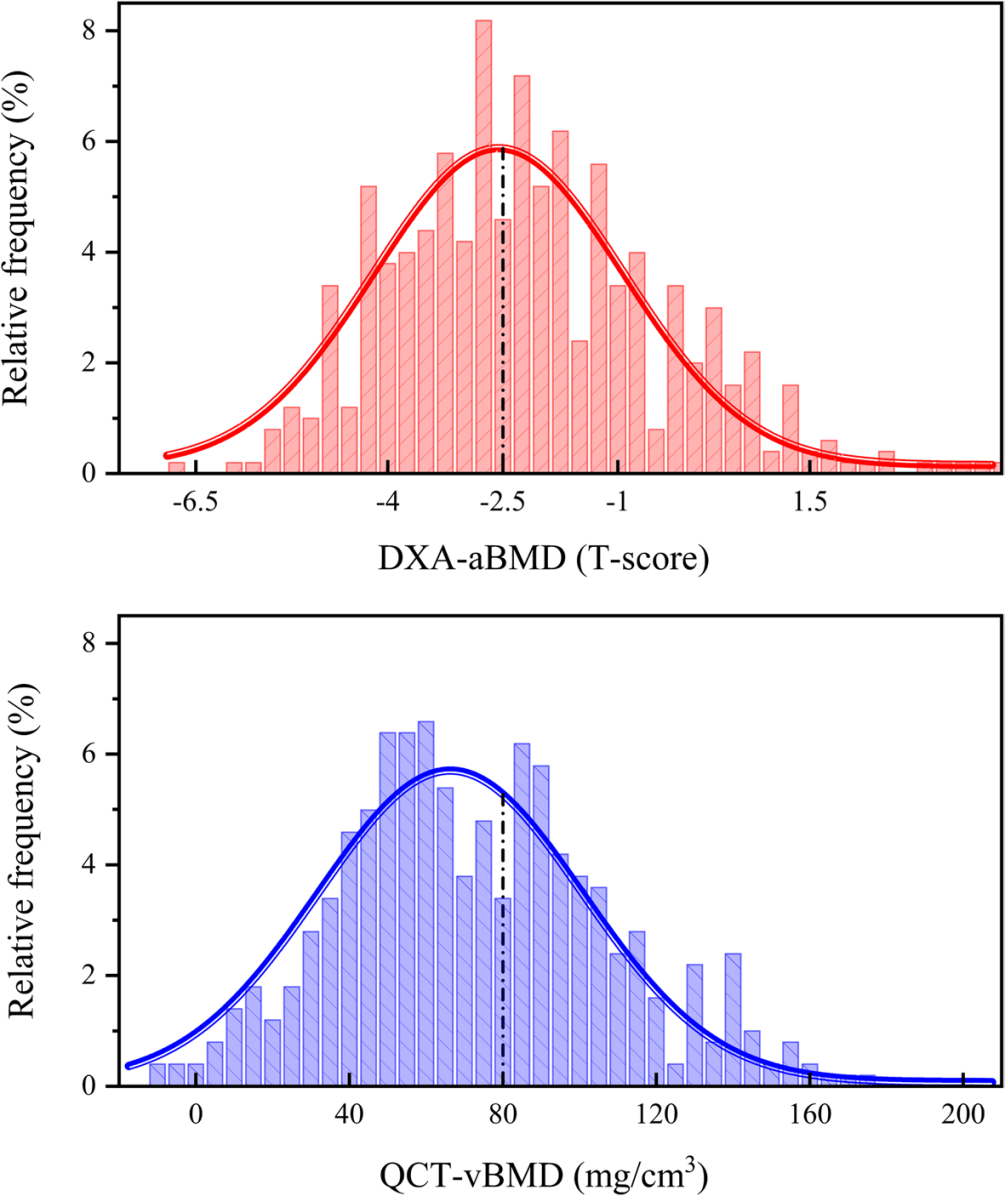
Curve fitting of BMD distribution (a bar plot superimposed with the probability density function) for all enrolled patients was performed using a nonlinear least-squares curve-fitting program with a Gaussian product function

Normality test by Kolmogorov‒Smirnov test showed that the distributions of data for QCT-vBMD and DXA T score are normal (P = 0.152 and 0.069, respectively). BMD distributions for QCT and DXA are plotted separately in Fig. 2. Both were relatively symmetric bell curves with similar distributional characteristics. The X-axis values relative to the peak of the curves of DXA T score distributions were near X = -2.5, while a similar point of the QCT-vBMD distribution curve was at the left of X = 80 mg/cm^3^. Similar results were also obtained in the subgroup of male and female patients (Supplementary Figures 1 and 2).

**Fig. 3:**
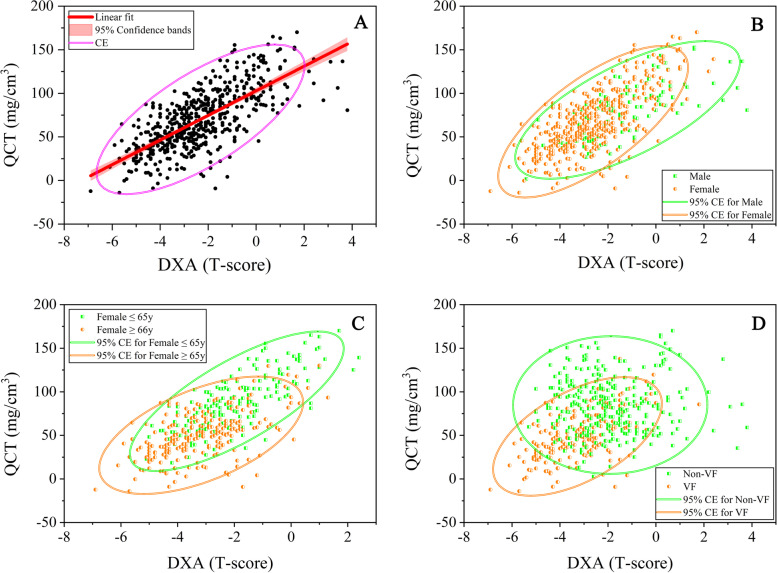
A scatter plot was drawn to demonstrate the relationship between QCT-vBMD (y-axis) and DXA T score (x-axis). The line of best fit for x against y is given by y = 14.1*x + 102.9, with a 95% CI of the slope ranging from 12.9 to 15.3 and R^2^ = 0.508 (**A**). **B**, **C**, and **D** indicate male versus female, age ≤ 65 years versus age ≥ 66 years, and VCF versus non-VCF

Scatter plots (Fig. 3) were drawn to analyze the correlations between QCT-vBMD and DXA T score, including the following subgroup analyses: sex, age stratification in postmenopausal women, and presence of VF. The line of best fit for DXA T score (x-axis) against QCT-vBMD (y-axis) was calculated as follows (Fig. 3 A): y = 14.1*x + 102.9, with a of slope from 12.9 to 15.3 and R^2^ = 0.508. The residual-versus-fitted plot, Supplementary Figure 3, shows that fitted values do not have an obvious trend of failure. Depending on different sex (Fig. 3 B), the line of best fit was y = 15.8*x + 108.7, R^2^ = 0.516 (female) and y = 11.7*x + 94.8, R^2^ = 0.478 (male). Depending on the age stratification in postmenopausal women (Fig. 3 C), the line of best fit was y = 16.1*x + 120, R^2^ = 0.573 (age ≤ 65 years) and y = 10.5*x + 83.5, R^2^ = 0.314 (age ≥ 66 years). Depending on the presence of VF (Fig. 3 D), the line of best fit was y = 12.7*x + 106.2, R^2^ = 0.451 (non-VF) and y = 10.6*x + 83.9, R^2^ = 0.308 (VF).

### Association between spinal BMD and VF

**Fig. 4:**
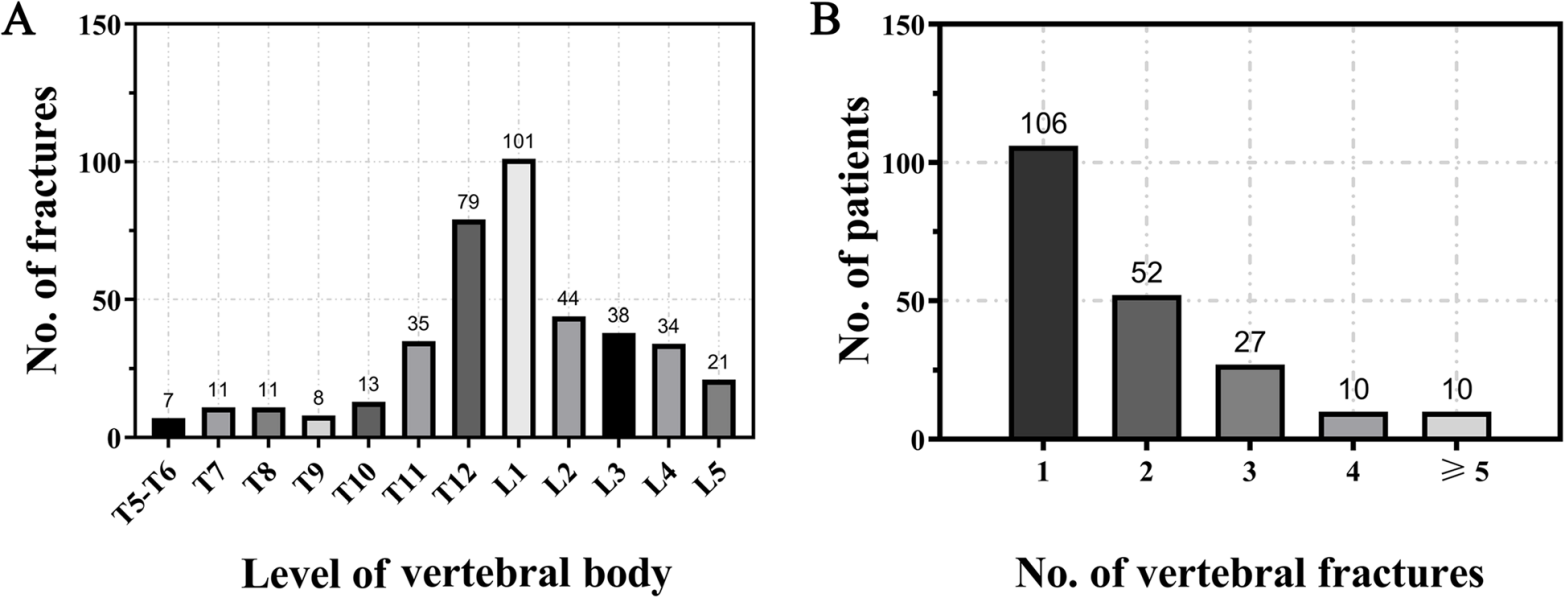
The distribution of fractured vertebrae at different levels (**A**). The distribution of the number of patients with single-level or multilevel VFs (**B**)

A total of 205 patients suffered a VF of at least one anatomic level. The VF occurred most commonly in the thoracolumbar spine: 101 fractures involved the L1 level and 79 involved the T12 level. Details regarding the VF status are shown in Fig. 4. Patients with VF had significantly lower BMD (49 mg/cm^3^ vs 84.9 mg/cm^3^, P < 0.01) and T-score (-3.25 vs. -1.67, P < 0.01) than those without VF. Among patients with VF, 84.4% (173/205) were diagnosed with osteoporosis by QCT, while only 73.2% (150/205) were diagnosed by DXA. A comparison of the baseline characteristics between the subgroups of patients with VF vs those without VF is shown in Table 3.

**Table 3:** Characteristics comparison between patients with VF versus without VF

**Variables**^a^	**Patients with VF** ***N* = 205**	**Patients without VF** ***N* = 296**	***P* value**
Female, n (%)	175 (85.4)	220 (74.3)	**0.003**
Age, year	72.5 ± 9.7	65.4 ± 9	**0.001**
Weight, kg	54.5 ± 9.9	59.3 ± 10	**0.001**
Height, cm	154.1 ± 7.2	156.2 ± 7.5	**0.001**
Body mass index, kg/m^2^	22.9 ± 3.6	24.3 ± 3.5	**0.001**
Steroid use, n (%)	14 (6.8)	7 (2.4)	**0.014**
Serum ionized calcium, mmol/L	2.27 ± 0.13	2.3 ± 0.14	0.026
Serum phosphorus, mmol/L	1.1 ± 0.17	1.12 ± 0.18	0.067
Serum uric acid, umol/L	293.3 ± 95.8	319.3 ± 102.1	**0.005**
Creatinine clearance, ml/min	62.6 ± 21.8	68.9 ± 23.6	**0.003**
25‐hydroxyvitamin D, ng/mL	29 ± 10.9	28.8 ± 11.3	0.862
Parathyroid hormone, pg/mL	46.1 ± 20.4	44.3 ± 20.8	0.385
Calcitonin, pg/mL	0.98 ± 1.91	0.94 ± 1.02	0.76
Alkaline phosphatase, U/L	83.2 ± 32.7	75.4 ± 23.6	**0.002**
P1NP, ug/L	68.7 ± 37.7	71.9 ± 34.9	0.558
CTX-1, ug/L	0.28 ± 0.28	0.3 ± 0.27	0.654
Hemoglobin A1c, %	6.2 ± 1.1	6.2 ± 1	0.786
Comorbidities, n (%)			
Cardiovascular disease, n (%)	90 (43.9)	139 (47)	0.499
Diabetes mellitus, n (%)	36 (17.6)	42 (14.2)	0.306
Respiratory disease, n (%)	21 (10.2)	17 (5.7)	0.061
Malignancy, n (%)	13 (6.3)	17 (5.7)	0.781
Cerebrovascular disease, n (%)	12 (5.9)	9 (3)	0.122
Chronic kidney disease, n (%)	10 (4.9)	8 (2.7)	0.198
DXA T-score	-3.25 ± 1.43	-1.67 ± 1.69	**0.001**
QCT-vBMD, mg/cm^3^	49 ± 27.3	84.9 ± 32	**0.001**
Osteoporosis diagnosis by DXA	150 (73.2)	104 (35.1)	**0.001**
Osteoporosis diagnosis by QCT	173 (84.4)	131 (44.3)	**0.001**

Bold values denote statistical significance. *P* < 0.05

*BMD* bone mineral density, *VF* vertebral fracture, *DXA* dual x-ray absorptiometry, *QCT* quantitative computed tomography, *P1NP* procollagen-1 N-terminal peptide, *CTX-1* C-terminal telopeptide of type-1 collagen

^a^Continuous variables were expressed as means and standard deviation; Categorical variables represent counts and frequencies

On the basis of univariate analysis, the factors that significantly correlated with VF status were further evaluated using multivariate logistic regression (Table 4). Collinearity analysis demonstrated no collinearity among the variables (i.e., all VIF < 1.1). Model 1 revealed that osteoporosis was an independent risk factors for VF when evaluated either by QCT or DXA. In the case of BMD evaluation using QCT, the OR value for osteoporosis was 6.81 (95%CI: 4.38–10.59), while the OR value for osteoporosis diagnosed by DXA was 5.04 (95%CI: 3.41–7.44). After adjusting for age, BMI, serum uric acid, creatinine clearance, and alkaline phosphatase in Model 2, the OR value for the osteoporosis by QCT and DXA were reduce to 3.44 (95% CI: 2.07–5.71) and 2.66 (95% CI: 1.68–4.21), respectively.

**Table 4:** Logistic regression analysis of risk factors associated with VCF

Variables	Patients with VCF (Model 1)			Patients with VCF (Model 2)		
	OR	95%CI	P	Adjusted OR^a^	95%CI	P
Osteoporosis by DXA	5.04	3.41–7.44	0.001	2.66	1.68–4.21	0.001
Osteoporosis by QCT	6.81	4.38–10.59	0.001	3.44	2.07–5.71	0.001

*VCF* vertebral compression fracture, *DXA* dual x-ray absorptiometry, *QCT* quantitative computed tomography

Bold values denote statistical significance. *P* < 0.05

^a^Model 2 was adjusted by age, gender, BMI, alkaline phosphatase, creatinine clearance, serum uric acid, and steroid use

**Fig. 5:**
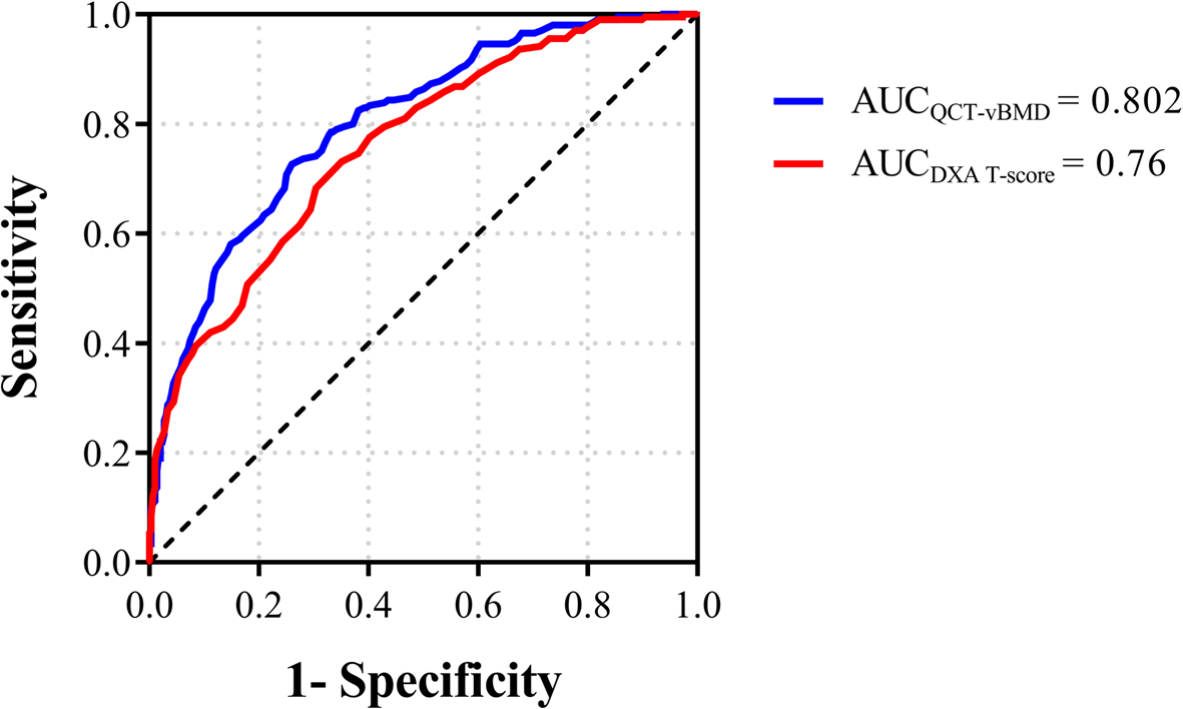
ROC plots for QCT-vBMD and DXA T score used for prediction of patients with prevalent VFs. **A**, **B**, and **C** indicate all patients, men, and women, respectively

For discrimination between patients with and without VF, QCT-vBMD (AUC = 0.802) showed better performance than DXA T score (AUC = 0.76) (Fig. 5). Similar VF identification results were found in the subgroup analysis of only female patients (AUC = 0.798 for QCT-vBMD vs. AUC = 0.748 for DXA T score) but not in the subgroup of older male patients (AUC = 0.779 for QCT-vBMD vs. AUC = 0.780 for DXA T score). The detailed ROC analysis is shown in Supplementary Table 3.

## Discussion

The present study involved a comparison between lumbar BMD measurements obtained by QCT-vBMD and by DXA T score in postmenopausal women and older men. Overall, BMD evaluation using QCT-vBMD resulted in identifying more patients with osteoporosis than that using DXA T score. In this cohort, using QCT enabled us to identify 10% more cases of osteoporosis than DXA (60.7% vs. 50.7%). A previous study in postmenopausal women with central obesity showed similar results, wherein spinal osteoporosis was found in 61.4% of women by QCT and in 47.7% of women by DXA [20]. The small sample size (44 postmenopausal women) limited further statistical analysis and generalization of the results. A recent age- and sex-matched study with a relatively large sample size reported that spinal osteoporosis detection rates for QCT were almost twice as high as those for DXA (58.16% vs. 30.63%), which is considerably different than the results in the present study[12]. Differences in the study population may be one of the reasons for this discrepancy; a higher prevalence of osteoporosis in patients undergoing spine surgery has previously been confirmed [21]. Another plausible explanation of the discrepancy may the different diagnostic criteria of spinal osteoporosis. A DXA T score = -2.5 diagnostic category recommended by the WHO was unreasonably applied in QCT; this situation, previously described in full, has been shown to result in a substantial overestimation of the diagnostic performance of QCT [2].

Despite the obvious overlap of ROI selection between the two technologies, there have been some inconsistent results regarding osteoporosis detection and VF prediction given the different imaging principles and diagnostic categories. A discordance in osteoporosis diagnoses between DXA and QCT was observed in 176 patients. A total of 72.7% (128/176) were detected with a lower BMD by QCT. A plausible explanation may be that bone loss is not uniform in age-related osteoporosis; trabecular bone is at a greater risk than cortical bone [22]. QCT can be used to quantify the vBMD of trabecular bone based on three-dimensional imaging while avoiding cortical bone interference. Moreover, limited by two-dimensional X-ray imaging, DXA-aBMD may be overestimated because of obesity, osteophytes, sclerosis, and aortic calcifications [23]. On these grounds, QCT may exhibit a higher sensitivity for BMD evaluation in patients with dominant cancellous bone loss. This interpretation is also supported by the BMD distribution curves (Fig. 2). After drawing a black reference line to mark the threshold for osteoporosis (dotted black line), we could easily see that the X-axis values relative to the peak of the curves of DXA T score distributions were near X = -2.5, while a similar point of the QCT-vBMD distribution curve was at the left of X = 80 mg/cm^3^. The osteoporosis detection rates of QCT initially peaked with decreased vBMD and then gradually declined with further lowering of vBMD, indicating a greater proportion of osteoporosis. In contrast, osteoporosis detection rates of DXA declined continuously with decreased BMD. This was similarly expressed in the level-specific BMD distribution [2]. The difference in these BMD distribution curves could also be explained by the age-related degeneration. With increasing age, systemic bone is lost gradually, especially cancellous bone; [22] at the same time, osteophytes, osteosclerosis, and abdominal aortic calcification gradually progress [24, 25]. In other words, older patients have lower BMD, more severe degeneration, and greater basis for DXA error, but QCT may not be affected by these factors.

The paucity of evidence-based data regarding the comparison between vBMD and T score distribution prompted us to perform linear correlation analysis. The model between QCT-vBMD as the reference method and DXA T score did not work very well (R^2^ = 0.508). To eliminate interference from confounding factors, such as age, sex, and VF status, linear regression of the scatter plots was performed for different subgroups, including male vs. female, age ≤ 65 years vs. ≥ 66 years, and with VF vs. without. However, performing additional subgroup analysis did not improve the goodness-of-fit of the regression models (R^2^ ranged from 0.308 to 0.516). Such a mismatch might not be explained by age, sex, and VF status. Future research should couple imaging characteristics at the measured ROI level with BMD data. Thus, it might be more helpful to explore the differences between QCT and DXA.

In most previous studies comparing diagnostic performance between QCT and DXA, the researchers have not investigated performance in identifying patients with and without VF. Our results revealed that osteoporosis detected by either QCT or DXA was an independent risk factor for prevalent VF (unadjusted/adjusted OR = 6.81/3.44 for QCT, unadjusted/adjusted OR = 5.04/2.66 for DXA). Using the multivariate model adjusted by as many factors as possible could lead to relatively lower ORs than models adjusted by age and sex only (OR from 4.02 to 6.9 for QCT-vBMD) [26, 27]. In other studies of any incident fractures instead of prevalent VFs, a lower OR is also a reasonable result (OR from 1.8 to 2.4 for QCT-vBMD) [28, 29]. Even though there were different OR values in the cited study, our results are in line with the findings in these studies; trabecular vBMD at the spinal lumbar region exhibited a stronger relationship with VF than lumbar DXA. QCT was more sensitive to age-related changes in vertebral body strength, which may justify its better performance for lumbar BMD evaluation in the older population.

Bone and muscle are the two major integrated components in the musculoskeletal system. With their complementary roles, both are vital for maintaining human health. A loss of skeletal muscle mass and function is considered an independent risk factor for fragility fractures as individuals so affected are at a higher risk of falling. The coexistence of sarcopenia and osteoporosis might ultimately worsen disability and health-related quality of life, especially for those characterized by physical frailty and functional impairment [30]. In oncology, the results of CT-based quantitative assessment of skeletal muscle are considered likely to be of prognostic value and be associated with postoperative complications, increased mortality, and overall survival in several cancers [31]. Both BMD and muscular quantity assessments should be integrated to optimize the comprehensive management of musculoskeletal health [32].

In the present study, discrimination between patients with and without VF based on QCT-vBMD was superior in relation to DXA T score (AUC = 0.802 vs. 0.76). It was reported from a recent study that vBMD obtained by routine CT compared to DXA can also improve the prediction of patients with prevalent VFs (AUC: 0.885 vs. 0.67). Another retrospective cohort study revealed the improved prediction of incident VF using opportunistic QCT compared to DXA (AUC: 0.76 vs. 0.63) [27]. The AUCs were different in the cited studies. However, owing to the heterogeneity of the study design, as well as differences in scanning protocols and study populations, any direct comparison between studies is not appropriate. Considering that BMD data obtained from the same individuals allowed for the elimination of influence from individual differences, this difference could be attributed to the advantage of the QCT technology itself. Studies in level-specific BMD evaluation also yielded similar results [2, 33].

The present study has several limitations. The study population was highly homogeneous, including only patients who were about to undergo spine surgery. Generalizability of the study results to patients from internal medicine services or other types of surgical practice may therefore be limited. Additionally, CT scans involve larger radiation doses and impose a greater financial burden on patients than DXA. However, unlike DXA, CT for patients scheduled for spine surgery is not restricted to BMD measurements and includes screening for other related disease and surgical planning. In future work, we should couple imaging characteristics at the measured ROI level with the BMD data. This approach might be more helpful in exploring the difference between QCT and DXA. Some importance should also be given to studying the effect of spinal-related degeneration and abdominal calcification on BMD evaluation, especially in patients classified with major discordance.

In conclusion, this study showed that preoperative QCT in older patients who undergo spine surgery is helpful to increase the spinal osteoporosis detection rate and improve the prediction of prevalent VF when compared to DXA.

## Supplementary Information

**Additional file 1: Supplementary Table 1.** Distribution of diagnostic category for lumbar BMD in female.

**Additional file 2: Supplementary Table 2.** Distribution of diagnostic category for lumbar BMD in male.

**Additional file 3: Supplemental Table 3. **AUCs with 95% CIs, Youden’s indices, and the resulting QCT/DXA thresholds and sensitivity and specificity values.

**Additional file 4: Supplementary Figure 1.** Curve fitting of BMD distribution for older men.

**Additional file 5: Supplementary Figure 2.** Curve fitting of BMD distribution for postmenopausal women.

**Additional file 6: Supplementary Figure 3.** The residual-versus-fitted plot shows that fitted values do not have an obvious trend of failure.

## Data Availability

The data that support the findings of this study are available from the corresponding author upon reasonable request (Zhiyun Wang, E-amil: Dragon201@126.com).

## Abbreviations

BMD: Bone mineral density
DXA: Dual-energy X-ray absorptiometry
QCT: Quantitative computed tomography
vBMD: Volumetric BMD
aBMD: Area BMD
VF: Vertebral fracture
ROI: Region of interest
OR: Odds ratio
CI: Confidence interval
ROC: Receiver operating characteristic
AUC: Area under the curve
