# Supplementary material for: Quantitative CT screening improved lumbar BMD evaluation in older patients compared to dual-energy X-ray absorptiometry
Source: BMC Geriatr. 2023 Apr 17;23:231. doi: 10.1186/s12877-023-03963-6 (PMC10108496; doi:10.1186/s12877-023-03963-6)
Supplement: Supplementary file 1 — Additional file 1: Supplementary Table 1. Distribution of diagnostic category for lumbar BMD in female. [file 12877_2023_3963_MOESM1_ESM.docx]

**Supplementary Table 1** Distribution of diagnostic category for lumbar BMD in female.

|  |  | QCT | | | |
| --- | --- | --- | --- | --- | --- |
|  |  | Normal | Osteopenia | Osteoporosis | Total |
| DXA | Normal | **29 (7.3%)** | 28 (7.1%) ^a^ | 7 (1.8%) ^b^ | 64 (16.2%) |
|  | Osteopenia | 5 (1.3%) ^a^ | **45 (11.4%)** | 54 (13.7%) ^a^ | 104 (26.3%) |
|  | Osteoporosis | 0^b^ | 31 (7.8%) ^a^ | **196 (49.6%)** | 227 (57.5%) |
|  | Total | 34 (8.6%) | 104 (26.3%) | 257 (65.1%) | 395 (100%) |

DXA, dual x-ray absorptiometry; QCT, quantitative computed tomography; a, minor discordance; b, major discordance.
