# Supplementary material for: Quantitative CT screening improved lumbar BMD evaluation in older patients compared to dual-energy X-ray absorptiometry
Source: BMC Geriatr. 2023 Apr 17;23:231. doi: 10.1186/s12877-023-03963-6 (PMC10108496; doi:10.1186/s12877-023-03963-6)
Supplement: Supplementary file 2 — Additional file 2: Supplementary Table 2. Distribution of diagnostic category for lumbar BMD in male. [file 12877_2023_3963_MOESM2_ESM.docx]

**Supplementary Table 2** Distribution of diagnostic category for lumbar BMD in male.

|  |  | QCT | | | |
| --- | --- | --- | --- | --- | --- |
|  |  | Normal | Osteopenia | Osteoporosis | Total |
| DXA | Normal | **11 (10.4%)** | 27 (25.5%) ^a^ | 7 (6.6%) ^b^ | 45 (42.5%) |
|  | Osteopenia | 0^a^ | **19 (17.9%)** | 15 (14.2%) ^a^ | 34 (32.1%) |
|  | Osteoporosis | 0^b^ | 2 (1.9%) ^a^ | **25 (23.6%)** | 27 (25.5%) |
|  | Total | 11 (10.4%) | 48 (45.3%) | 47 (44.3%) | 106 (100%) |

DXA, dual x-ray absorptiometry; QCT, quantitative computed tomography; a, minor discordance; b, major discordance.
