# Supplementary material for: Quantitative CT screening improved lumbar BMD evaluation in older patients compared to dual-energy X-ray absorptiometry
Source: BMC Geriatr. 2023 Apr 17;23:231. doi: 10.1186/s12877-023-03963-6 (PMC10108496; doi:10.1186/s12877-023-03963-6)
Supplement: Supplementary file 3 — Additional file 3: Supplemental Table 3. AUCs with 95% CIs, Youden’s indices, and the resulting QCT/DXA thresholds and sensitivity and specificity values. [file 12877_2023_3963_MOESM3_ESM.docx]

**Supplemental Table 3** AUCs with 95% CIs, Youden’s indices, and the resulting QCT/DXA thresholds and sensitivity and specificity values.

| **Test variable** | **State variable** | **AUC** | **95%CI** | **THRE** | **SENS** | **SPEC** | **YI** |
| --- | --- | --- | --- | --- | --- | --- | --- |
| QCT vBMD (mg/cm^3^) | VF | 0.802 | 0.763-0.84 | 61.5 | 0.727 | 0.74 | 0.467 |
| DXA T-score | VF | 0.76 | 0.718-0.802 | -2.45 | 0.732 | 0.649 | 0.38 |

AUC, area under the curve; CI, confidence interval; THRE, threshold of VBQ score; SENS, sensitivity; SPEC, specificity; YI, Youden’s index; DXA, dual X-ray absorptiometry; QCT, quantitative computed tomography; vBMD, volumetric bone mineral density; VF, vertebral fracture.
